# Isolation and characterization of an algicolous endophytic *Mucor circinelloides* from macroalga *Colpomenia sinuosa* as a promising source of bioactive metabolites for dermocosmetic applications

**DOI:** 10.1038/s41598-026-63698-3

**Published:** 2026-08-16

**Authors:** Mostafa M. El-Sheekh, Nessma A. El-Zawawy, Ebrahim M. Shalamesh, Alshaimaa R. Elsaadawy, Yehia A.-G. Mahmoud

**Affiliations:** Department of Botany and Microbiology, Faculty of Science, Tanta University, Tanta, 31527 Egypt

**Keywords:** Marine endophytes, *Colpomenia sinuosa*, Antibacterial activity, Tyrosinase inhibition, Molecular docking, Cosmeceuticals, Biochemistry, Biotechnology, Drug discovery, Microbiology

## Abstract

Marine endophytic fungi have arisen as a prospective source of bioactive secondary metabolites with potential cosmeceutical and pharmaceutical applications. In this study, four endophytic fungal isolates were obtained from Egyptian marine algae *Colpomenia sinuosa*. Preliminary screening for antibacterial activity against *Staphylococcus epidermidis* indicated that the isolate EF2 was the most active, exhibiting an inhibition percentage of 40.67%, and therefore was selected for optimization by the Plackett–Burman experiment and response surface methodology. This optimization significantly improved the inhibitory activity from 40.67% to 94.4%. The optimized crude extract of isolate EF2 exhibited strong antioxidant activity with the DPPH assay and significant anti-inflammatory activity by cyclooxygenase-2 (COX-2) enzyme inhibition. Furthermore, it demonstrated pronounced tyrosinase inhibitory activity, suggesting its potential role in melanin biosynthesis regulation. The isolate was molecularly identified as *Mucor circinelloides* AUMC 16435 with accession number PP837661. Cytotoxicity assessment revealed selective activity against carcinoma cells with relatively low toxicity toward normal fibroblasts, indicating a favorable safety profile. GC–MS analysis of EF2 isolate crude extract (*Mucor circinelloides* AUMC 16435) demonstrated several bioactive constituents, with 3-methoxy-2, 4, 6-trimethyl-cyclohex-2-enone and 2-fluoro-3-trifluoromethylbenzoicacid-3-hexadecyl ester as the predominant compounds. Molecular docking studies further confirmed favorable binding interactions of the compounds within the tyrosinase active site, with energies of binding -6.1 and -5.9 kcal/mol, respectively. Overall, this study reports the first isolation of *M. circinelloides* from Egyptian *C. sinuosa* and highlights its potential as a promising source of bioactive metabolites with enhanced antibacterial activity and multifunctional properties suitable for pharmaceutical and dermocosmetic applications.

**Supplementary Information:**

The online version contains supplementary material available at 10.1038/s41598-026-63698-3.

## Introduction

Endophytes are a varied group of microorganisms, including bacteria and fungi, that live symbiotically inside plant tissues without causing harm^1,2^. These mutualistic associations provide a suitable habitat and nutrients for endophytic microorganisms. In return, the host benefits from enhanced resistance to pathogens and pests, together with improved tolerance to environmental stresses such as salinity and drought^3^. Notably, many endophytes have the ability to produce various secondary metabolites with biological potential that contribute to host defense mechanisms. Endophytic microorganisms gained significant interest because of their ability to synthesize structurally diverse bioactive compounds with potential applications in industry, medicine, and agriculture^4,5^. There are multiple biological activities, such as antioxidant, anticancer, and antibacterial properties, that have been demonstrated by these metabolites, making endophytes a promising source for natural product discovery.

Endophytes are not limited to terrestrial plants but are also associated with aquatic organisms, including marine algae. Many studies have been reported on bacterial and fungal endophytes isolated from seaweeds, along with the identification of new bioactive compounds, particularly those with antimicrobial properties^5–9^. Despite these advances, research on marine algal endophytes in Egypt remains relatively limited and is restricted to a small number of host species. In particular, there is a clear lack of information regarding endophytic microorganisms associated with *C. sinuosa*. To date, no comprehensive studies have explored the diversity or biological activities of endophytes derived from this marine alga. This gap emphasizes how crucial it is to look into *C. sinuosa* as an unexplored source of endophytic fungi that can produce new bioactive compounds.

Skin aging is a complex process influenced by both intrinsic factors, including oxidative stress and metabolic changes, and extrinsic factors, including ultraviolet (UV) radiation^10–13^. Prolonged UV exposure accelerates skin aging and contributes to structural damage, pigmentation disorders, inflammation, and increased risk of skin cancer^14^. Consequently, there is growing interest in developing cosmeceutical products incorporating natural antioxidants and photoprotective agents to mitigate these effects^10,13,14^. In addition, tyrosinase plays a key role in melanin biosynthesis, and its inhibition represents an effective strategy for controlling hyperpigmentation and enhancing skin-brightening effects^15,16^. Furthermore, maintaining skin homeostasis requires controlling microbial imbalance, as microorganisms such as *Staphylococcus epidermidis* and *Malassezia* species are associated with various skin disorders, including inflammatory acne^17^. Therefore, natural compounds exhibiting combined antioxidant, antimicrobial, anti-inflammatory, and antityrosinase activities are highly desirable for dermocosmetic applications.

To the best of our knowledge, this is the first study reporting the isolation of *Mucor circinelloides* as an endophytic fungus from the marine alga *Colpomenia sinuosa*. In addition to characterizing the isolate, the present work provides the first comprehensive evaluation of its optimized antibacterial metabolite production, antioxidant, anti-inflammatory, anti-tyrosinase, cytotoxic, and dermocosmetic-related activities, supported by GC–MS profiling and molecular docking analysis.

## Material and methods

### Collection of *Colpomenia sinuosa* and isolation of endophytic fungi (EF)

**Fig. 1:**
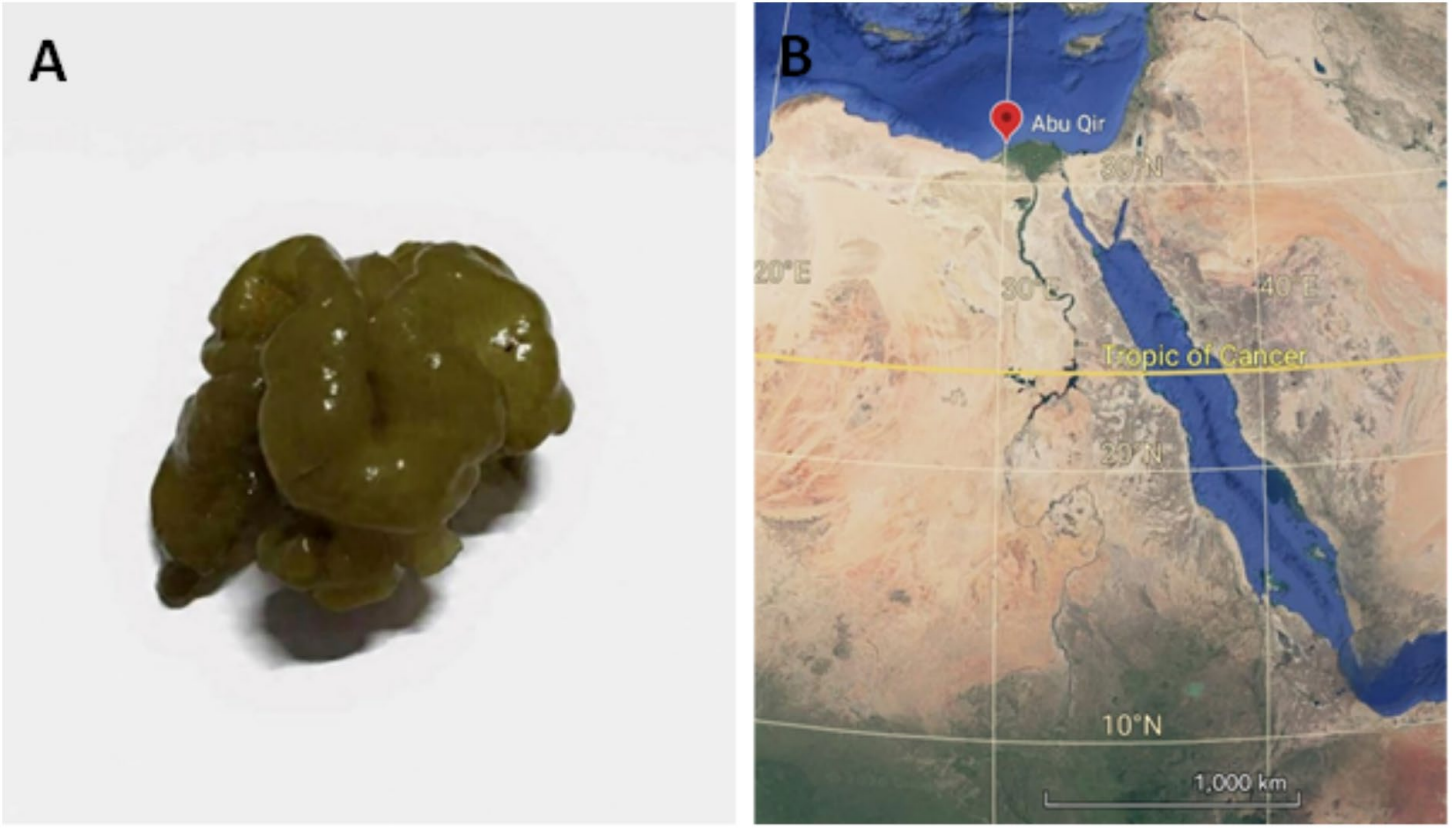
(**A**) *Colpomenia sinuosa* collected from Abu Qir, Alexandria, Egypt. (**B**) Map showing the geographic location of the sampling site on the Mediterranean coast of Abu Qir, Alexandria, Egypt.

*Colpomenia sinuosa* attached fresh thalli were randomly collected from Abu Qir, Alexandria, Egypt, in May 2023 (Fig 1). Taxonomic identification of *C. sinuosa* was done according to Aleem^18^ and the Algae Base website’s for identification and habitat data used to confirm^19^.

The voucher specimen (*Colpomenia sinuosa* Herb. Nasr-Br4-3/5/2023) has been deposited at the herbarium of the late Professor Abdel-Halim Nasr at the Botany and Microbiology Department, Faculty of Science, Alexandria University, Egypt. The collection and the deposition of the voucher specimen were carried out with the help of Prof. Dr. Mohamed S. Abdel-Kareem, professor of phycology, Department of Botany and Microbiology, Faculty of Science, Alexandria University, Egypt. Collecting samples of seaweed didn’t require any specific permission, as the sampling beaches aren’t protected areas, and *C. sinuosa* is neither an endangered nor vulnerable species. The algal samples were quickly sent to laboratory. All samples were thoroughly rinsed under running tap water and cut into approximately 0.5-cm segments. The segments were then washed three times with sterile seawater to remove attached debris. Surface sterilization was performed by immersion in 70% ethanol for 60–120 s, followed by 4% sodium hypochlorite for 60 s. Finally, the samples were rinsed with sterile distilled water for 10 s before plating^20^. After being semi-dried on sterile tissue paper, samples were put on top of a Petri plate containing Sabouraud Dextrose Agar (SDA) medium supplemented with chloramphenicol, according to Zuccaro et al.^21^ and Zhang et al.^22^. The plates were incubated at 28 °C in the dark and examined daily for the emergence of fungal colonies from the surface-sterilized algal tissues. Four endophytic fungal isolates designated as (EF1, EF2, EF3 and EF4) were obtained and repeatedly moved onto new plates with SDA until pure cultures were obtained. Each isolate of endophytic fungi was subcultured into three new SDA plates for the next steps.

### Small-scale fermentation and extraction of secondary metabolites

The fermentation experiment on a small scale was done according to the study of Kjer et al.^23^ , with some modifications. Specifically, four mycelial plugs (6 mm) from each fungal sample were obtained from its growing edges, and in 100 mL of Sabouraud Dextrose Broth (SDB) mycelial plugs were inoculated and incubated at 28 ˚C for 15 days with shaking (140 RPM) in dark conditions. The metabolites generated by each fungus were extracted using ethyl acetate (EtOAc)^24^. Broth culture was filtered by using a funnel and filter paper, Whatman No. 1 to separate the mycelia. After the extraction of the filtrate using an equal amount of ethyl acetate (1:1) three times, it was allowed to stand for twenty minutes, and then the organic solvent phase was separated. Using a rotary evaporator, the organic phase was concentrated at reduced pressure. The crude extract was stored at -20 °C for further use.

### Assessment of bioactive compounds activity by antimicrobial assay

Endophytic fungi crude extracts were diluted in ethyl acetate (200 μg/mL) and assessed against *Staphylococcus epidermidis* ATCC 25923 for antibacterial activity to estimate production of bioactive compounds. Agar well diffusion experiment was done as reported by El-Zawawy et al.^25^ , with some modifications. Plates of nutrient agar were inoculated with 100 μL bacterial suspension (10^6^ CFU/mL). One hundred μL of each extract were loaded into 8 mm-wide hole in the center of the nutrient plates, and then were incubated for 24 h at 37 °C. After incubation, zones of inhibition around wells appeared, indicating the bioactivity of each crude extract. Inhibition zones were measured, and inhibition percentages were calculated according to Horta et al.^26^

The solvent only was the negative control, while vancomycin (5 µg/mL) was the positive control^27^. The microdilution technique was used to evaluate the minimum inhibitory concentration (MIC) with resazurin dye^28^. The existence of live cells was indicated by the resazurin dye’s color change from pink to red, which made growth observation possible. Bacterial growth was totally suppressed, as evidenced by the dark blue. The lowest concentration of the sample that prevented the growth of bacteria was used to estimate MIC.

### Experimental designs for optimization of bioactive compounds production for the most potent EF isolate

#### Plackett-Burman (PB) experimental design

The Plackett-Burman (PB) experimental design was utilized to identify key factors impacting bioactive compound production in the chosen isolate. Five variables reflecting the elements of the fermentation conditions and basal medium at high (+1) and low (-1) levels were assessed, as explained in Table S1. Thirteen runs were ordered with the Plackett-Burman design. To avoid mistakes, there are three duplicate center points in this design matrix. All tests were duplicated with 250 mL Erlenmeyer flasks, and the average of the duplicates was used to determine the final result. The average inhibitory percentage (%) of bioactive substances was used to calculate the response^29^. Determination of the first-order model statistical significance was done by Fisher’s test for analysis of variance (ANOVA)^30^. The base that the Plackett-Burman design depends on is the first-order model:

1 $${\mathrm{Y}} = \, \beta 0 \, + \sum \beta {\text{i Xi}}$$

Where Y is the dependent variable or response (inhibition percentage); β0 is the model intercept; βi is the linear coefficient; and Xi is the level of the independent variable.

#### Response surface methodology using Box-Behnken design (BBD)

To enhance bioactive metabolites production, the Plackett-Burman experiment was used to recognize the three most important parameters that enhance bioactive compound production, which were then adjusted using the Box-Behnken design. These variables were analyzed at three levels: high (+1), intermediate (0), and low (-1), as explained in Table S2. There were seventeen trials in the experiment. Using 250 mL Erlenmeyer flasks, each experiment was performed in triplicate, and the average of the duplicates was used to calculate the final response value. An equation of second-order polynomial was used to analyze the results. The equation of general formula for a second-order polynomial is:

2 $${\text{Y }} = {\text{ }}\beta _{0} - {\text{ }}\sum \beta _{{\mathrm{j}}} {\mathrm{X}}_{{\mathrm{j}}} + \sum \beta _{{{\mathrm{ii}}}} {\mathrm{X}}_{{\mathrm{j}}} ^{{\mathrm{2}}} + {\text{ }}\sum {\text{ }}\beta _{{{\mathrm{ij}}}} {\mathrm{X}}_{{\mathrm{i}}} {\mathrm{X}}_{{\mathrm{j}}}$$

Where Y is the predicted response; β*i* is the linear coefficient, β0 is the intercept term, β*ii* is the interaction coefficient, β*ij* is the quadratic coefficient, and *Xi Xj* represent the independent variables^31^.

The program "Design Expert Software" (Version 7.0) was used to do regression analysis on the collected data, and confirmed by Microsoft Excel 2016^32^. The polynomial model equation precision was assessed using R^2^ and adjusted R^2^. The response surface three-dimensional (3D) graphs were employed to represent the equation of the fitted polynomial. The optimal value of each variable for the highest response was found using the Excel solver and the Design Expert numerical optimization method.

### DPPH (2, 2-Diphenyl-1-picrylhydrazyl) radical scavenging assay

Determination of antioxidant activity of a crude extract for selected EF isolate was performed by the DPPH radical scavenging method. A fresh DPPH solution (0.004% w/v) in methanol was prepared and stored at 10 °C in the dark until use. The extract was diluted in methanol in (0.02, 0.2, 2, 20, and 200 µg/mL) concentrations. Mixtures of 3 mL of the DPPH solution and 40 µL of each concentration were prepared. Immediately after mixing, at 515 nm the absorbance was measured using a spectrophotometer UV-visible (Milton Roy; Spectronic 1201). Readings were taken at one-minute intervals until no further change in absorbance was seen (about 16 minutes)^33,34^. A DPPH solution was negative control, and the positive control was ascorbic acid. All experiments were done in triplicate, and analysis was done using the average values. The radical scavenging activity percentage was calculated as:

3 $${\text{Antioxidant activity }}\left( \% \right) \, = \, \left[ {\left( {{\mathrm{A}}_{{\mathrm{C}}} - {\mathrm{A}}_{{\mathrm{T}}} } \right) \, /{\text{ A}}_{{\mathrm{C}}} } \right] \, \times { 1}00$$

Where A_C_ = Control absorbance at t = 0 min and A_T_ = Absorbance of the sample + DPPH at t = 16 min.

### Cytotoxicity assay

Cytotoxic potential of crude extract for selected EF isolate was evaluated against cell lines of human epidermoid carcinoma (A431) and normal human skin fibroblast (HSF) which were provided from the American Type Culture Collection (ATCC, Rockville, MD, USA). The assay was done at Al-Azhar University, in the Regional Center for Mycology and Biotechnology (RCMB). A431 cells were cultivated in RPMI-1640 medium with gentamycin (50 µg/mL) and 10% heat-inactivated fetal calf serum. HSF cells were cultured in DMEM with HEPES buffer, 10% heat-inactivated fetal bovine serum, 1% L-glutamine, and gentamycin (50 µg/mL). To guarantee exponential growth, cells were subcultured twice a week in a 5% CO2 humidified atmosphere at 37 °C. For the cytotoxicity assay, 96-well plates were inoculated with 100 µL of cells per well, and the cells were given 24 hours to adhere^35^. The cells were then treated with a range of extract concentrations (0.02–200 µg/mL). After treatment, cell viability was evaluated by the absorbance measurement, and the percentage of viable cells was determined compared to the control. Negative, vehicle, and positive controls were the cells fed with DMEM alone, 1% DMSO, and 10% DMSO, respectively. The viability and cytotoxicity percentage of cells were determined as:

4 $${\text{Viability }}\left( \% \right) \, = {\text{ Absorbance of treated sample }}/{\text{ Absorbance of untreated }} \times { 1}00$$

$${\text{Cytotoxicity }}\left( \% \right) \, = { 1}00{-}{\text{Viability }}\left( \% \right)$$

### *In-vitro* photoprotective assay

The crude extract of the selected EF isolate at (25, 50, 100, 150, and 200 μg/mL) concentrations was analyzed using a spectrophotometer UV-visible from 290 to 320 nm at 5 nm intervals. The sun protection factor (SPF) was determined according to the equation of Mansur^36^:

5 $${\text{SPF = }}\,{\mathrm{CF}}\,\sum\nolimits_{{290}}^{{230}} {{\mathrm{EE}}} (\lambda )\,\,{\mathrm{I}}(\lambda )\,\,{\mathrm{abs}}(\lambda )$$

Where CF (correction factor) is 10; I(λ) is solar light intensity at λ wavelength; EE (λ) is the erythema effect of solar radiation at λ wavelength; and abs (λ) is spectrophotometric absorbance values of samples at λ wavelength; The values of EE(λ) I(λ) are obtained from the correlation between EE and I at each λ wavelength.

### *In vitro* anti-inflammatory activity

Inhibition of cyclooxygenase-2 (COX-2) enzyme is a technique that was used to obtain the anti-inflammatory activity of the selected EF isolate crude extract. The assay was performed based on a standard enzymatic method with minor adjustments^37^. The COX-2 enzyme was incubated with concentrations of extract (0.02, 0.2, 2, 20, and 200 µg/mL) for five minutes at room temperature (25 °C) with hematin present. Arachidonic acid (50 µM), leucodichlorofluorescein (20 µM), hematin (1 µM), and phenol (500 µM) were pre-mixed in 1 mL of Tris buffer (pH 8, 0.1 M) to create the reaction mixture. This combination was then mixed with the enzyme-sample solution, which started the reaction. Enzymatic activity was measured by a spectrophotometer UV-visible (Milton Roy; Spectronic 1201) by detecting changes in the absorbance at 502 nm over 1 minute^38^. A blank sample without enzyme and Celecoxib as a negative and positive control, respectively.

### Skin-aging-related tyrosinase enzyme inhibitory assay

Tyrosinase enzyme inhibition percentage was evaluated by performing the mushroom tyrosinase inhibition test^39^. Eighty microliters of phosphate buffer (pH 6.8, 0.1 M) was combined with 40 μL of L-tyrosine in a 96-well plate then incubated for 10 minutes at 37 °C. Various concentrations of crude extract for selected EF isolate (0.02, 0.2, 2, 20, 200 μg/mL in 1% DMSO) were applied to the wells with mushroom tyrosinase (250 U/mL in phosphate buffer saline (PBS)). The absorbance was measured by an ELISA reader (Multiskan Sky High; Thermo Fisher Scientific) at 475 nm at 60s intervals over 120 minutes. Ascorbic acid is positive control and PBS instead of a sample was control. Inhibition percentage of tyrosinase enzyme was calculated as:

6 $${\text{Tyrosinase inhibition }}\left( \% \right){\text{ }} = {\text{ }}\left[ {\left( {{\mathrm{A}}_{0} - {\text{ A}}_{{\mathrm{1}}} } \right){\text{ }}/{\text{ A}}_{0} } \right]{\text{ }} \times {\mathrm{1}}00$$

Where A_0_ is the control absorbance; A_1_ is the sample absorbance.

### Identification of the most potent EF isolate

The ITS-rDNA sequence of the selected isolate EF2 was compared with homologous sequences available in the GenBank database using the BLASTn algorithm of the National Center for Biotechnology Information (NCBI) to identify closely related taxa. Representative ITS sequences of *Mucor circinelloides* and related *Mucor* species were retrieved from GenBank and aligned using MEGA version 12.1.2^40^. Phylogenetic analysis was performed using the Maximum Likelihood (ML) method based on the Tamura–Nei nucleotide substitution model^41^. The robustness of the inferred phylogenetic tree was evaluated using 1000 bootstrap replicates^42^. *Rhizopus arrhizus* CBS112.07 (GenBank accession No. NR_103595) was used as the outgroup. The resulting phylogenetic tree was used to confirm the taxonomic position of isolate EF2 within the genus *Mucor*.

### Gas chromatography-mass spectrum (GC-MS) analysis

Chemical profiling of the EF2 crude extract was performed at the Scientific Research Center and Measurements (SRCM), Tanta University, Tanta, Egypt, using a PerkinElmer Clarus 580 gas chromatograph coupled to a Clarus 560S mass spectrometer and equipped with an Elite-5MS capillary column (30 m × 0.25 mm i.d., 0.25 μm film thickness). A 1 μL aliquot of the crude extract was manually injected into the GC–MS system. The oven temperature was initially maintained at 60°C for 10 min, followed by heating at a rate of 10°C min⁻^1^ to 260°C, where it was held for 5 min. The total chromatographic run time was 35 min, and the solvent delay was set to 6 min. Mass spectra were acquired in electron ionization (EI) mode over an m/z range of 50–600 Da. Compound identification was achieved by comparing the obtained mass spectra with those in the National Institute of Standards and Technology (NIST) Mass Spectral Library, and the relative abundance of each constituent was estimated from the corresponding peak area percentage as previously described with minor modifications^43,44^.

### Molecular docking analysis

The main compounds found, 2-fluoro-3-trifluoromethylbenzoic acid-3-hexadecyl ester and 3-methoxy- 2, 4, 6-trimethyl-cyclohex-2-enone, were analyzed for their binding affinities to the tyrosinase enzyme using molecular docking. Avogadro software (version 1.2.0) was employed to optimize the geometry of the selected ligands after their chemical structures were obtained from the DrugBank database. Tyrosinase’s 3D structure was obtained from the RCSB Protein Data Bank (PDB ID 3NM8). AutoDockTools (version 1.5.7) was used to get the protein structure ready for docking^45^, which included removing water molecules and non-essential heteroatoms, assigning Kollman charges, adding polar hydrogen atoms, and correcting missing atoms. The prepared protein was then saved in pdbqt format.

The active site was defined based on the binding pocket residues, and a grid box was generated to fully enclose the catalytic region. 0.375 Å was used as the grid spacing. AutoDock Vina was used to conduct docking simulations^46^. Resulting interactions between the target protein and the ligands, and binding modes were further investigated and visualized using Biovia Discovery Studio software (version 21.1.0.20298).

## Results and discussion

### Macroalgal sampling and isolation of endophytes

Endophyte presence and variation in various algae are most likely influenced by various abiotic factors as light, temperature, salinity, and nutrient pollution^47^. Several investigations have found changes in endophyte diversity based on algal host genus^48,49^. Therefore, investigating endophytic fungi from different marine macroalgae and habitats remains an important approach for identifying promising microorganisms capable of producing valuable secondary metabolites.

**Fig. 2:**
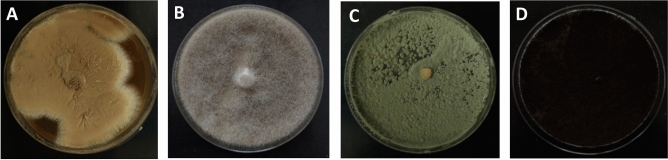
Isolated endophytic fungi genera from *C. sinuosa*. **A**: EF1: *Aspergillus* sp*.*1. **B**: EF2: *Mucor* sp. **C**: EF3: *Penicillium* sp*.* **D**: EF4: *Aspergillus* sp*.*2.

The current study aimed to study the various activities of endophytes isolated from collected Egyptian *C. sinuosa* for the first time, which was obtained from Abu Qir, Alexandria, Egypt. Four fungal endophytes were obtained from the collected algae and were identified morphologically at the genus level, as in Fig. 2. These endophytes were distinct from those isolated from the same algae at various places ^50^. The observed diversity of endophytic fungi is consistent with previous reports describing marine algae as important reservoirs of diverse endophytic microorganism^51^. The isolation of diverse fungal endophytes from *C. sinuosa* further supports the growing recognition of marine algae as promising yet underexplored sources of microorganisms capable of producing biologically active secondary metabolites.

### Antimicrobial assay

**Fig. 3:**
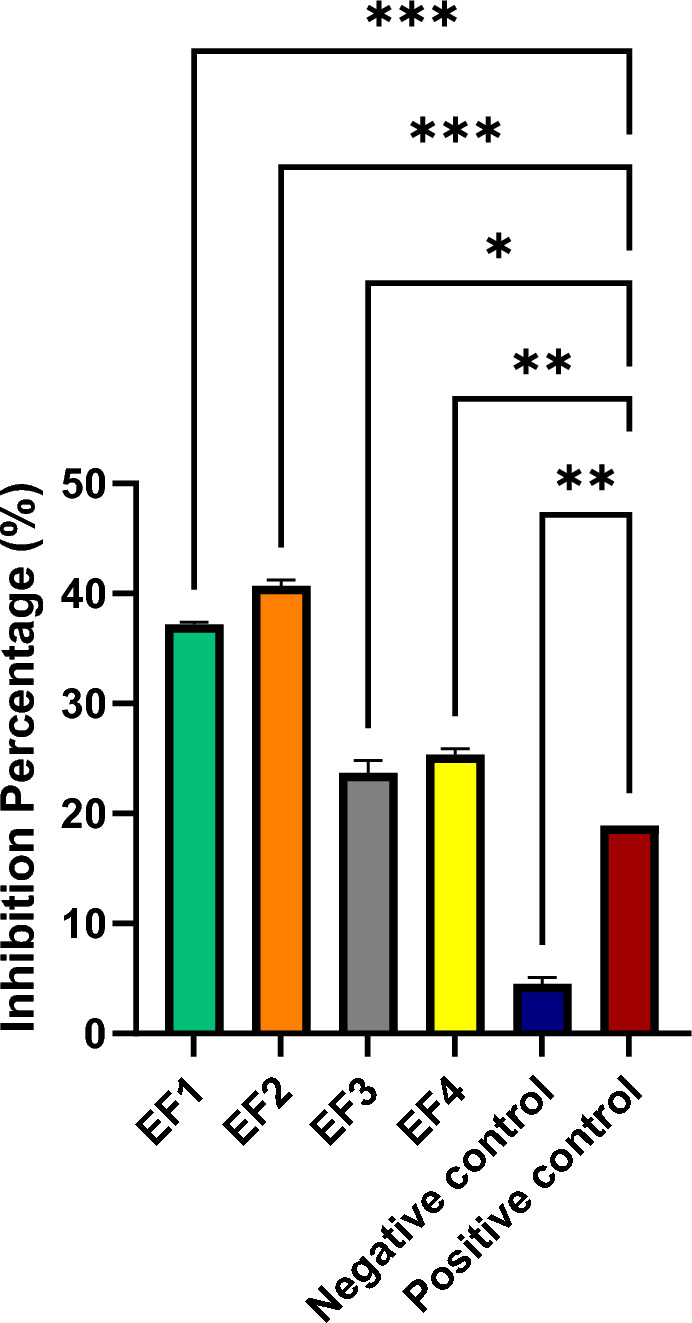
Antibacterial activity of isolated endophytic fungi (EF1, EF2, EF3, and EF4) from *C. sinuosa.*

The four isolated endophytic fungi (EF1, EF2, EF3, and EF4) crude extracts were assessed for their ability to inhibit *S. epidermidis* pathogen *in vitro* as shown in Fig. 3. EF2 showed the highest inhibition activity against *S. epidermidis* 40.67 % and was classified as the highest active endophyte; While EF1, EF3, and EF4 had moderate inhibitory activity compared to positive and negative controls. EF2 also gave the lowest MIC value against *S. epidermidis* gram-positive bacterium, of 15.6 µg/mL. The remarkable antibacterial activity of EF2 may be attributed to its ability to produce extracellular secondary metabolites that interfere with bacterial growth or cellular metabolism. Marine-derived endophytic fungi are increasingly recognized as prolific producers of antimicrobial metabolites, reflecting their ecological role in protecting the algal host against microbial competitors and environmental stress^51^. Similarly, the study of Kaur and Arora^52^ showed that an isolated endophytic *Chaetomium* sp. from *Moringa oleifera* gave antimicrobial efficiency against the used strain. Based on these promising results, EF2 was selected for subsequent optimization to maximize the production of its bioactive metabolite.

### Optimization of bioactive compounds production from crude extract of EF2

It has been established that fungal endophytes are a potential source of bioactive substances. Optimization of fungal fermentation conditions can help cost-effectively produce these compounds^29^. In comparison to the traditional procedure "one factor at a time", the optimization process with experimental design techniques is more effective and competitive^53^. In this optimization method, the first step was to build up a rapid screen for fermentation parameters to determine which had a substantial influence on the process of bioactive compound production. This was preserved using the Plackett-Burman design (PBD). PBD is much simpler and consumes less time than other statistical methods^54^. This technique was used to get the optimal values of the variables affecting the generation of bioactive compounds from EF2 crude extract.

**Fig. 4:**
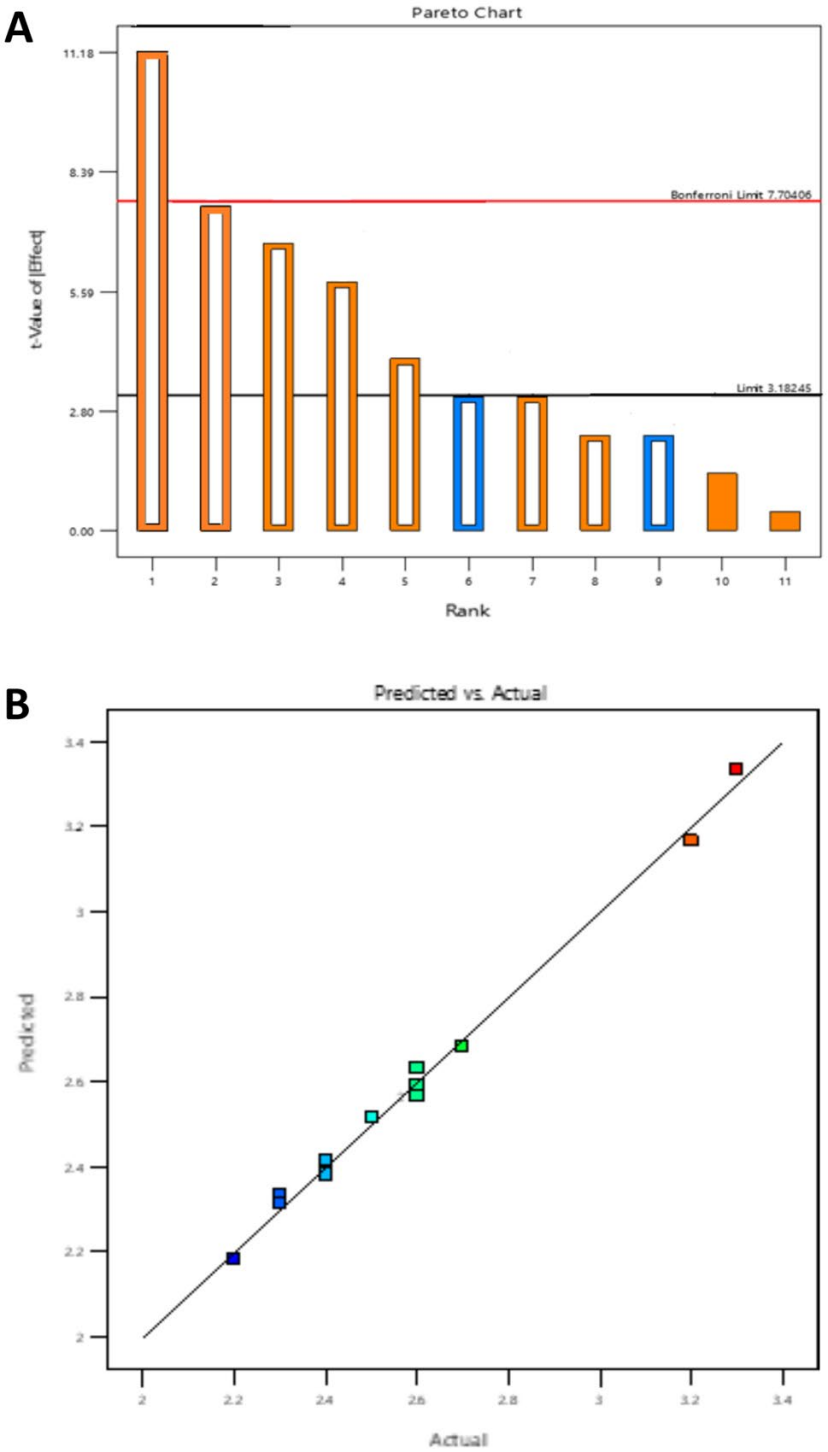
Pareto chart depicts the degree to which each variable influences bioactive compounds production by EF2 isolate (**A**). Correlation between the experimentally actual and predicted values for bioactive compounds production by EF2 isolate according to the Plackett–Burman experimental results (**B**).

To evaluate the factors with a significant effect on the bioactive compounds produced from crude extract of EF2, a PBD design was used with an inhibitory percentage of *S. epidermidis* as a response. Table 1 shows that thirteen variables were studied and their impacts on bioactive compounds production. The PBD findings indicated that the inhibition percentage of *S. epidermidis* varied from 8.5% to 85.4%. According to the Pareto chart (Fig. 4A); peptone was the most significant factor influencing inhibition percentage, followed by glucose and temperature. The model’s applicability was validated by the predicted vs. actual inhibition percentage plot (Fig. 4B), which showed that the experimental findings almost matched the theoretical results supplied by the model equation. Table 2 explained the statistical analysis’s findings. The impact of each component on the *S. epidermidis* inhibition percentage was determined using the P-value. The model’s fitness was determined using the determination coefficient, or R^2^. The model showed 99% of the response variability, according to the R^2^ value of 0.993. Thus, in this study, the model offered a reliable estimate of the bioactive compounds production. The model was very significant, according to the adjusted coefficient of determination value (Adj.R^2^=0.9644). The model’s F-value of 34.16 indicated that it was significant after calculation of the experimental design analysis of variance (ANOVA). Peptone, glucose, and temperature were shown to be the most effective factors influencing the inhibition percentage of *S. epidermidis* based on PBD results. These parameters were chosen for additional optimization by the Box-Behnken experiment and the response surface approach.

**Table 1:** Plackett-Burman design matrix for evaluating significant factors affecting bioactive compounds production by EF2 isolate.

**Run**	**Peptone**	**Glucose**	**Temperature**	**Incubation period**	**Inoculum size**	**Inhibition percentage (%)**	
						**Actual value**	**Predicted value**
1	1	1	-1	-1	-1	85.2	69.40
2	1	-1	-1	-1	1	32.6	42.70
3	1	-1	1	1	1	12.6	18.43
4	1	-1	1	1	-1	8.5	2.77
5	1	1	1	-1	-1	9.3	15.13
6	1	1	-1	1	1	30.4	30.40
7	0	0	0	0	0	85.4	91.10
8	-1	1	-1	1	1	12.6	7.97
9	-1	-1	-1	1	-1	16.7	11.23
10	-1	1	1	-1	1	21.3	21.20
11	-1	1	1	1	-1	23.4	18.90
12	-1	-1	-1	-1	-1	35.4	34.07
13	-1	-1	1	-1	1	9.4	19.50

**Table 2:** Analysis of variance (ANOVA) and regression statistics for the experimental results of Plackett Burman design of bioactive compounds production by EF2 isolate.

**Source**	**Sum of Squares**	**df**	**Mean square**	**F-value**	**p-value**	
**Model**	1.28	9	0.1423	34.16	0.0288	significant
A-peptone	0.5208	1	0.5208	125.00	0.0079	
B-glucose	0.0675	1	0.0675	16.20	0.0565	
C-temp	0.0408	1	0.0408	9.80	0.0887	
D-Incubation period	0.1875	1	0.1875	45.00	0.0215	
E-inoculum size	0.0208	1	0.0208	5.00	0.1548	
AD	0.2408	1	0.2408	57.80	0.0169	
BD	0.1408	1	0.1408	33.80	0.0283	
Curvature	0.0001	1	0.0001	0.0154	0.9126	
**Residual**	0.0083	2	0.0042			
**Cor Total**	1.29	12	R- Squared	0.9935		
**Std. Dev.**	0.0645		Adj R-Squared	0.9644		
**Mean**	2.59		Adeq Precision	19.3677		
**C.V%**	2.49					

**Fig. 5:**
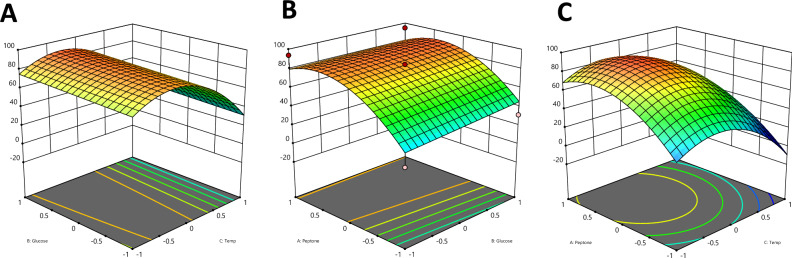
The 3D surface response showing the mutual interaction effect of peptone, glucose, temperature on bioactive compounds production by EF2 isolate.

In order to determine the levels needed for the applied factors to obtain the response value desired in a small number of tests, the Response Surface Method (RSM) was utilized^55^. This approach was also used to examine the interaction of many factors^56^. The Box Behnken Design (BBD) of RSM has been shown in several studies to be a dependable and efficient tool for developing and optimizing a variety of processes^57^. As seen in Table 3, the BP experiment was planned and executed with the most efficient settings. The Design Expert software was utilized to submit the resulting data to ANOVA. The results of a t-test used to evaluate the polynomial model’s statistical significance are shown in Table 4. According to our results, the quality of the model was high, which meant that it could effectively explain how the different medium components interacted. The findings demonstrated that the model met appropriate standards and could describe the actual interaction between medium components. The main effects of each parameter on inhibition percentage, as well as their interactions, 3D response surface, and contour graphs were used to illustrate them. Fig. 5 shows the 3D response surface graphs for significant pairwise combinations of the three variables. Each 3D response surface plot’s z-axis was used to plot the inhibition percentage against two independent parameters that were examined concurrently, with the third parameter remaining constant at zero (an intermediate value), as seen in Fig. 5a, B, And C.

**Table 3:** Seventeen trials of Box-Behnken design representing bioactive compounds production by EF2 isolate.

**Run**	**Peptone**	**Glucose**	**Temperature**	**Inhibition percentage (%)**		**Residual**
				**Actual value**	**Predicted value**	
1	0	0	0	83.2	83.70	-0.5000
2	1	0	-1	35.9	68.78	-32.88
3	-1	0	1	18.7	-8.23	26.93
4	-1	1	0	31.4	45.65	-14.25
5	-1	-1	0	21.4	35.50	-14.10
6	1	0	1	35.6	31.07	4.53
7	-1	0	-1	30.9	29.47	1.43
8	1	1	0	94.4	80.30	14.10
9	0	1	-1	91.7	75.42	16.28
10	0	0	0	84.3	83.70	0.6000
11	0	-1	1	16.9	32.22	-15.32
12	0	1	1	21.6	37.72	-16.12
13	0	0	0	84.4	83.70	0.7000
14	1	-1	0	93.7	79.45	14.25
15	0	-1	-1	85.1	69.92	15.17
16	0	0	0	83.3	83.70	-0.4000
17	0	0	0	83.3	83.70	-0.4000

**Table 4:** ANOVA of the fitted quadratic polynomial model of bioactive compounds production by EF2 isolate.

**Source**	**Sum of squares**	**df**	**Mean square**	**F-value**	**p-value**	
**Model**	12474.71	7	1782.10	0.0213	0.0109	significant
A-Peptone	3088.98	1	3088.98	0.0218	0.0108	
B-Glucose	60.50	1	60.50	0.7073	0.4520	
C-Temperature	2842.58	1	2842.58	0.0262	0.0177	
AB	21.62	1	21.62	0.8219		
A^2^	2327.74	1	2327.74	0.0396		
B^2^	0.0059	1	0.0059	0.9970		
C^2^	3767.40	1	3767.40	0.0136		
Residual	3623.42	9	402.60			
Lack of Fit	3622.00	5	724.40	< 0.0001		significant
Pure Error	1.42	4	0.3550			
Cor Total	16098.13	16	R^2^	0.9935

**Fig. 6:**
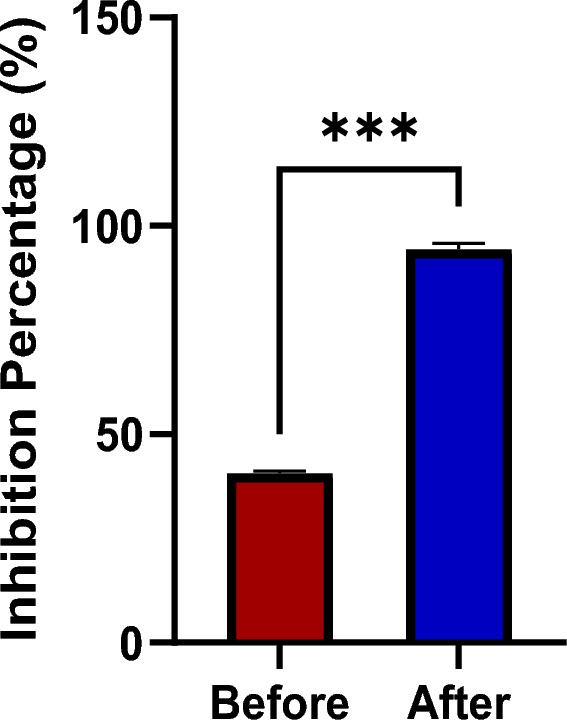
Inhibition percentage of EF2 isolate before and after RSM optimization.

The best conditions for the highest inhibition percentage were obtained to be 30 g/L glucose, 15 g/L peptone, and at 28 °C, with the use of BBD numerical optimization. Compared to the response before the whole optimization stage, the inhibition percentage increased 2.32-fold from 40.67% to 94.4% after the numerical optimization strategy was applied. (Fig. 6). This finding aligned with the study of Salem et al.^58^ , which showed increasing production of laccase from endophyte *Trichoderma harzianum* by the response surface method. Moreover, the response surface method causes a 1.49-fold improvement in the production of resveratrol by *Arcopilus aureus* endophyte^59^.

### Antioxidant and cytotoxicity activities

The results of antioxidant activity performed by the optimized EF2 crude extract are given in Table 5 and Fig. S1. Crude extract of isolate EF2 showed a significant IC_50_ value 1.42 μg/mL in comparison to IC_50_ value 0.32 μg/mL of ascorbic acid in DPPH radical scavenging method. The strong free radical scavenging activity suggests that EF2 produces a diverse array of antioxidant secondary metabolites capable of neutralizing reactive oxygen species (ROS), which are major contributors to oxidative stress and skin aging. These findings corroborated those of Calado et al.^60^ , who reported that the marine endophyte *Halopteris scoparia* contained bioactive compounds with potential dermocosmetic uses. In addition, Khalil et al.^61^ showed *Chaetomium* sp. endophytic fungi and their antioxidant activities. Furthermore, Banji-Onisile et al.^62^ reported that fungal endophytic fungi from *Terminalia phanerophlebia* showed antioxidant, antimicrobial, and cytotoxic activities. The potent antioxidant activity observed in the present study is consistent with previous reports demonstrating that crude extracts of endophytic fungi can exhibit remarkably low IC_50_ values owing to the synergistic effects of multiple bioactive secondary metabolites, including phenolics, terpenoids, and polyketides^63–65^. Since oxidative stress plays a pivotal role in the initiation of inflammation, photoaging, and hyperpigmentation, the pronounced antioxidant activity of the EF2 crude extract provides a strong biological basis for its subsequent evaluation as an anti-inflammatory and anti-tyrosinase agent.

**Table 5:** Antioxidant activity of optimized EF2 crude extract compared to ascorbic acid.

	Antioxidant activity (%)	
Conc. (μg/mL)	EF2	Ascorbic acid (Control)
0.02	9.61±0.86	14.48±0.62
0.2	21.30±0.57	43.01±2.87
2	53.09±1.67	79.35±1.22
20	73.64±1.17	94.25±0.92
200	87.02±1.63	98.6±0.10
**IC**_**50**_	1.42	0.32

Each value corresponds to the mean ± SD (n = 3).

**Fig. 7:**
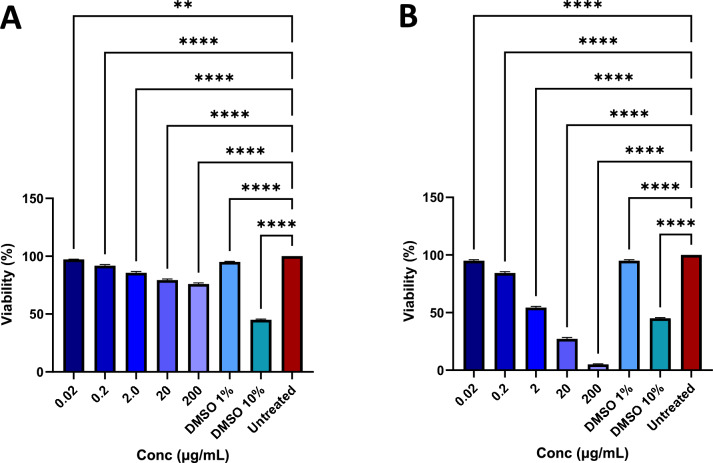
Cytotoxicity evaluation of optimized EF2 crude extract against HSF (**A**) and A431 (**B**).

Furthermore, cytotoxicity screening is a vital part of creating safe and effective medications ^66,67^. Through this study, *In vitro* A431 and HSF cell lines were employed to evaluate the cytotoxic activity of optimized EF2 crude extract using the MTT assay. Fig 7A and B show the cytotoxic impact of EF2 on normal and tumor cell lines. The study determined that EF2 was nontoxic to the HSF cell line, enabling normal cell growth and metabolism (IC_50_ more than 200 μg/mL). as in Fig. 7A. These findings demonstrated that EF2 is a very safe biocompatible substance for normal cells, since it did not affect the normal cell line. This favorable safety profile is particularly important for the potential development of topical dermocosmetic formulations, where minimal toxicity toward normal skin cells is a fundamental requirement. Whereas EF2 was able to reduce tumor cell viability in a dosage-dependent manner, as in Fig. 7B, long-time exposure resulted in increased cell toxicity. The IC_50_ for isolate EF2 was 3.7 μg/mL (Fig S2). As a result, EF2 has been considered to be a natural anti-tumor agent. These findings almost agree with the study of Sahoo et al.^20^ , who revealed that *Biscogniauxia petrensis*, a marine endophytic fungi, had bioactive efficacy against different cell lines of cancer. Furthermore, Taritla et al.^68^ isolated *Aspergillus* sp*.* as an algal–derived endophytic fungus that produced cytotoxic secondary metabolites. The selective cytotoxic activity observed in this study is also in agreement with previous investigations reporting that crude extracts from endophytic fungi possess potent antiproliferative activity against a variety of human cancer cell lines while exhibiting lower toxicity toward normal cells, supporting their potential as a source of safe bioactive compounds^69,70^.The combination of potent antioxidant activity, selective cytotoxicity toward tumor cells, and low toxicity toward normal fibroblasts further highlights the multifunctional nature of the EF2 crude extract and supports its potential for future pharmaceutical and dermocosmetic applications.

### Sunscreen protection factor (SPF) and anti-inflammatory activity

**Fig. 8:**
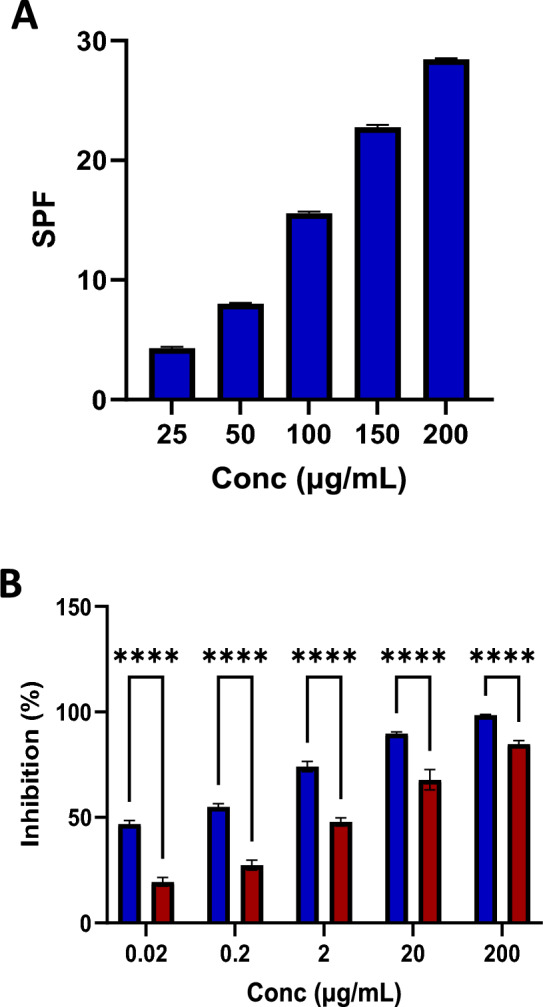
*In-vitro* sun protection factor (SPF) of optimized EF2 crude extract (**A**) and anti-inflammatory activity of optimized EF2 crude extract (**B**). The results expressed as mean ± SD (n = 3).

The optimized EF2 crude extract has performed a high SPF value of 28.5 (Fig 8A), indicating that it may be helpful for skin protection from the damaging effects of UV radiation when using as a sunscreen. FDA recommends SPF 15 sunscreens for protection from skin cancer, sunburn, and quick aging of the skin^71^. As a result, EF2 isolated crude extract showed promise for future sunscreen and personal care product formulations, with less negative effects than organic sunscreens^72^. EF2 crude extract can disperse UVR and absorb UV light as a physical barrier. In addition, it may act as an absorbent filter that reduces UV radiation penetration through the epidermis. The observed photoprotective activity may be attributed to the presence of antioxidant secondary metabolites capable of scavenging UV-induced reactive oxygen species, thereby minimizing oxidative damage to skin cells. Similarly, the study of Maciel et al.^73^ showed the photoprotective ability of secondary metabolites from *Annulohypoxylon stygium* algae-associated fungi. Moreover, studies showed fungal endophytes from *Colobanthus quitensis,* which enhance photoprotective mechanisms^74^. The combination of high antioxidant activity and elevated SPF value suggests that EF2 may provide dual protection against both direct UV exposure and oxidative stress-mediated skin damage, making it particularly attractive for dermocosmetic applications.

Cyclooxygenase enzymes (COXs) are key oxidoreductases that catalyze the conversion of arachidonic acid into prostaglandin H₂, a central precursor of multiple pro-inflammatory mediators included in the regulation of inflammatory responses^75^. In the current study, the anti-inflammatory potential of optimized EF2 crude extract was evaluated through the inhibition of COX-2 *in vitro*. COX-2 activity was significantly inhibited by the tested extract, with an IC_50_ 4.648 µg/mL, compared to celecoxib, which showed an inhibition with an IC_50_ 1.7 µg/mL (Fig. 8B and Fig S3). Although less potent than the standard drug, the observed COX-2 inhibition highlights the extract’s ability to interfere with key inflammatory pathways. The suppression of cyclooxygenase activity suggests that the extract may contribute to the modulation of inflammatory processes, supporting its potential application in managing inflammatory skin conditions. Oxidative stress and inflammation are closely interconnected biological processes, as excessive reactive oxygen species can stimulate COX-2 expression and promote the production of inflammatory mediators^76^. Therefore, the strong antioxidant activity demonstrated by isolate EF2 may contribute, at least in part, to its observed anti-inflammatory effect. These findings are almost agree with the study that demonstrated the anti-inflammatory properties of endophytic *Fusarium solani* against COX enzymes^77^, while metabolites derived from *Aspergillus versicolor* endophytes have shown notable anti-inflammatory and antioxidant effects^78^. Collectively, the combined antioxidant, photoprotective, and COX-2 inhibitory activities suggest that EF2 possesses a multifunctional biological profile that may help protect the skin against UV-induced inflammation and photoaging, further supporting its potential use as a natural dermocosmetic ingredient.

### Antiaging activity through tyrosinase enzyme inhibition

The melanogenesis process in humans and the enzymatic browning in fruits and fungi are naturally occurring processes controlled by the tyrosinase enzyme^79^. Tyrosinase mediates the tyrosine oxidation into dopaquinone, which is the first step in melanogenesis. Dopaquinone is then converted by autooxidation to L-dopachrome and L-DOPA. Tyrosinase uses L-DOPA as a substrate before melanin is formed^80^. Tyrosinase and melanosome synthesis are increased by sun exposure, which causes hyperpigmentation. As a result, the cosmetics industry has observed a rise in demand for natural and safe tyrosinase inhibitors as skin-whitening treatments^81^. In addition to regulating skin pigmentation, tyrosinase inhibition has become an important strategy in modern dermocosmetics because excessive melanogenesis is closely associated with photoaging and UV-induced skin damage^76^. Therefore, natural tyrosinase inhibitors with multifunctional biological activities are increasingly preferred over synthetic depigmenting agents due to their improved safety profile^82^**.**

Table 6 summarizes the tyrosinase inhibitory activity of optimized EF2 crude extract in comparison with ascorbic acid at different concentrations. At a concentration of 200 µg/mL, both EF2 and ascorbic acid exhibited strong inhibitory effects, reaching 98.1 ± 0.21% and 99.8 ± 0.1%, respectively.

**Table 6:** Results of anti-aging related tyrosinase inhibition percentage of optimized EF2 crude extract compared to positive control.

	Tyrosinase inhibition (%)	
Conc (μg/mL)	EF2	Ascorbic acid (Control)
0.02	35.2 ± 0.44	59.2 ± 0.46
0.2	41.5 ± 1.11	68.9 ± 0.82
2	64.2 ± 0.6	81.8 ± 0.36
20	82.4 ± 0.69	97.4 ± 0.25
200	98.1 ± 0.21	99.8 ± 0.1

Values expressed as mean ± SD (n = 3).

The observed activity of EF2 crude extract is almost consistent with previous reports highlighting the tyrosinase inhibitory potential of endophytic fungi. Similarly, *Fusarium solani* was previously identified as a source of substances capable of inhibiting tyrosinase activity^77^. Moreover, Dwibedi et al.^59^ reported that the endophyte *Arcopilus aureus* possesses a pronounced property of tyrosinase inhibition. In another study, it was demonstrated by Calado et al.^60^ that the marine endophyte *Halopteris scoparia* and its metabolites exhibit significant tyrosinase inhibition with potential applications in dermocosmetics. Furthermore, a review by Devi et al.^83^ emphasized the role of endophytic fungi as promising sources of tyrosinase inhibitors. Given its combined antioxidant and tyrosinase inhibitory activities, the isolate EF2 extract may offer a dual functional role in cosmetic formulations by mitigating oxidative damage associated with skin aging and regulating melanin production, highlighting its efficacy as an anti-aging and natural skin-whitening agent, supported by the molecular docking analysis, which demonstrated favorable interactions between the major identified metabolites and the active site of the enzyme.

### Molecular identification

**Fig. 9:**
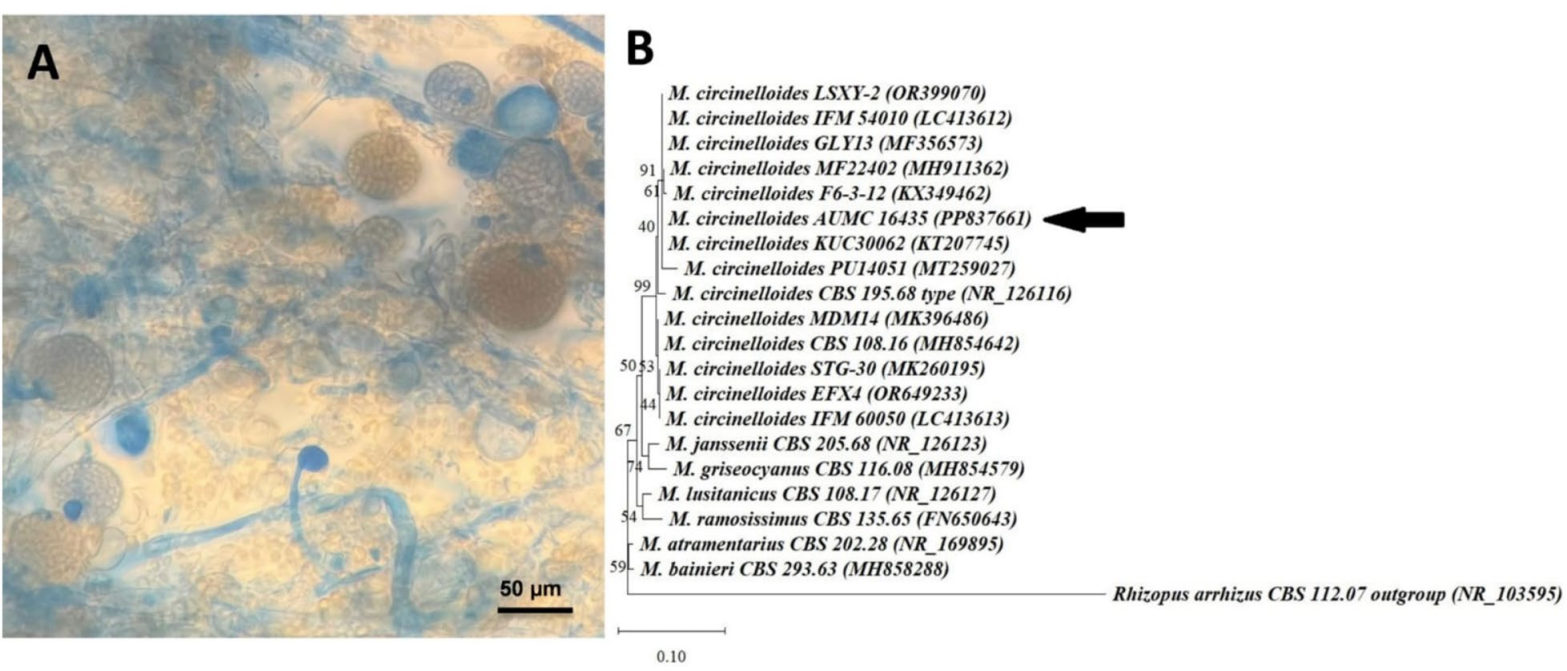
Morphological and molecular identification of EF2 isolate. Microscopic examination of EF2 isolate at 40x magnification (**A**)**.** A phylogenetic tree was generated using ITS sequences of rRNA of the EF2 (*Mucor circinelloides* AUMC16435; GenBank accession number PP837661) (**B**).

The selected endophytic isolate (EF2) was molecularly identified as *Mucor circinelloides* based on ITS rDNA sequence analysis and was deposited in GenBank under accession number PP837661. The phylogenetic relationship between EF2 and closely related reference strains is presented in Fig. 9, confirming its taxonomic placement within the genus *Mucor*. To the best of our knowledge, this is the first report describing *M. circinelloides* as a marine endophytic fungus isolated from the Egyptian brown alga *Colpomenia sinuosa***.** This finding expands the current knowledge of the endophytic fungal diversity associated with *C. sinuosa* and highlights this marine alga as a promising yet underexplored source of bioactive fungal endophytes. The remarkable biological activities exhibited by the isolate EF2 crude extract further emphasize the potential of this newly identified marine endophyte as a valuable source of secondary metabolites for future pharmaceutical and dermocosmetic applications.

### GC–MS analysis of EF2 isolate crude extract

The chemical components of optimized EF2 crude extract were profiled by GC-MS analysis. Several substances were found, such as 2-fluoro-3-trifluoromethylbenzoic acid-3-hexadecyl ester, 3-methoxy-2, 4, 6-trimethyl-cyclohex-2-enone, 1-nonadecene, 1-hexadecene, and 4-isobenzofuranol, octahydro-3a, 7a-dimethyl-(3aα, 4β, 7aα), as summarized in Table 7 and Fig. S4. Based on peak regions and retention periods, the GC-MS chromatography showed the relative abundance of the identified components. Among the identified compounds, 2-fluoro-3-trifluoromethylbenzoic acid-3-hexadecyl ester and 3-methoxy-2, 4, 6-trimethyl-cyclohex-2-enone were the most predominant components in the extract. Numerous biological responses, such as anti-inflammatory, antidiabetic, antioxidant, antibacterial, and anticancer properties, have been previously documented for identified metabolites, in addition to potential applications in pharmaceutical and cosmetic formulations^84–89^.Although GC–MS successfully identified the predominant volatile and semi-volatile constituents of the EF2 crude extract, the broad spectrum of biological activities observed is likely attributable to the synergistic effects of multiple metabolites, including minor or non-volatile constituents that may not be efficiently detected by GC–MS analysis. Therefore, future studies involving bioassay-guided fractionation coupled with advanced LC–MS/MS-based metabolomic approaches and molecular networking will be valuable for comprehensive metabolite characterization and for identifying the specific compounds responsible for the observed biological activities.

**Table 7:** GC/MS analysis for optimized EF2 isolate crude extract.

Compound name	Formula	Structure	Mol. Wt (g/mol)	Area (%)	RT
3-Methoxy-2,4,6-trimethyl-cyclohex-2-enone	C₁₀H₁₆O₂		168.23	45.762	21.084
2-Fluoro-3-trifluoromethylbenzoic acid, 3-hexadecyl ester	C₂_4_H₃_6_F₄O₂		432.5	6.419	24.976
1-Nonadecene	C₁₉H₃₈		266.49	5.151	27.642
1-Hexadecene	C₁₆H₃₂		224.42	4.584	22.485
4-Isobenzofuranol, octahydro-3a,7a-dimethyl-, (3aα,4β,7aα)	C₁₀H₁₈O_2_		170.252	3.840	21.650

### Molecular docking study

**Fig. 10:**
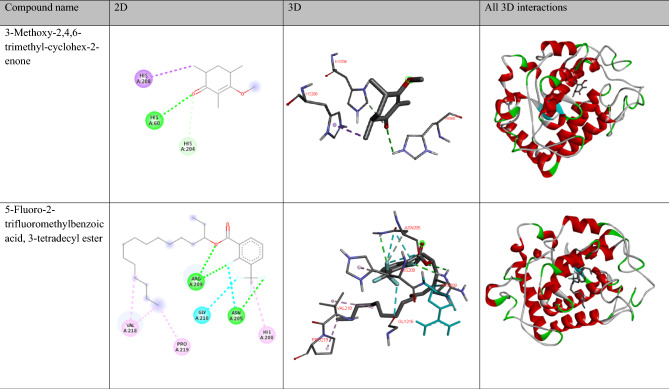
Molecular docking of the major bioactive compounds of optimized EF2 isolate crude extract with tyrosinase protein represented in 2D and 3D conformation.

Molecular docking is a computational method that predicts the best binding orientation and affinity of ligands toward target proteins, providing valuable insights into molecular interactions at the active site^45^. The interaction between major bioactive compounds and the tyrosinase active site was evaluated using molecular docking. Both two-dimensional (2D) and three-dimensional (3D) interaction profiles were analyzed to better understand ligand–receptor binding patterns, as in Fig. 10. The results showed that both compounds had good affinity for binding to the enzyme. Specifically, 2-fluoro-3-trifluoromethylbenzoic acid-3-hexadecyl ester had a binding energy -5.9 kcal/mol, whereas 3-methoxy-2, 4, 6-trimethyl-cyclohex-2-enone demonstrated a slightly stronger interaction with a binding energy -6.1 kcal/mol. Detailed interaction analysis revealed that 2-fluoro-3-trifluoromethylbenzoic acid-3-hexadecyl ester established hydrogen bonding interactions with ASN205 and ARG209 within the active site, in addition to multiple hydrophobic contacts with surrounding residues. In contrast, 3-methoxy-2, 4, 6-trimethyl-cyclohex-2-enone formed hydrogen bonds with HIS60 and HIS204, which are key residues in the catalytic region of the enzyme. Although the first compound exhibited a greater number of hydrogen bond interactions, the second compound displayed a more favorable binding energy, suggesting enhanced binding stability within the tyrosinase active site. A summary of docking scores, hydrogen bond distances, and interacting residues is provided in Table S3. Importantly, the localization of these interactions within the catalytic pocket supports the experimentally observed tyrosinase inhibitory activity. This finding is aligned with previous studies showing that strong binding interactions with key active site residues, particularly histidine residues, are closely associated with effective tyrosinase inhibition^90,91^. Therefore, the molecular docking results should be interpreted as supportive and hypothesis-generating rather than definitive evidence of biological activity. Further bioassay-guided isolation and experimental validation of the active metabolites are required to confirm their direct contribution to the observed biological activities.

Collectively, the biological activities observed in the present study are unlikely to result from a single metabolite but rather from the synergistic action of multiple secondary metabolites identified in the crude extract. The combined antioxidant, anti-inflammatory, antimicrobial, and anti-tyrosinase activities, together with the favorable safety profile and docking results, highlight the multifunctional nature of the extract and support its potential as a promising candidate for dermocosmetic and pharmaceutical applications.

## Conclusion

This study represents the first report of isolating the fungal endophyte *M. circinelloides* from the Egyptian marine alga *C. sinuosa*, identifying a previously unexplored source of bioactive metabolites. The crude extract of *M. circinelloides* exhibited significant anti-inflammatory, antioxidant, and tyrosinase inhibitory activities. These bioactivities were further supported by GC–MS profiling, while molecular docking analysis provided preliminary evidence of favorable interactions between the major identified compounds and the tyrosinase active site. However, these *in silico* findings should be considered supportive rather than conclusive and require further experimental validation. Moreover, EF2 showed selective cytotoxicity against carcinoma cells while exerting relatively low toxicity toward normal cells, suggesting a favorable safety profile. Overall, these findings highlight the *M. circinelloides* extract as a promising candidate for pharmaceutical and dermocosmetic applications, warranting further bioassay-guided isolation, *in vivo* evaluation, and formulation development to confirm the active metabolites, safety, and efficacy.

## Data availability.

The dataset generated and/or analyzed during the current study is available in the GenBank repository under accession number PP837661. https://www.ncbi.nlm.nih.gov/nuccore/PP837661.

## Supplementary Information

Supplementary Information.
