# Supplementary material for: Isolation and characterization of an algicolous endophytic Mucor circinelloides from macroalga Colpomenia sinuosa as a promising source of bioactive metabolites for dermocosmetic applications
Source: Sci Rep. 2026 Aug 16;16:25533. doi: 10.1038/s41598-026-63698-3 (PMC13478274; doi:10.1038/s41598-026-63698-3)
Supplement: Supplementary file 1 [file 41598_2026_63698_MOESM1_ESM.doc]

Supplementary

Table S1. Experimental independent variables at three levels affecting bioactive compounds production isolate using Plackett Burman design

| Variables | Symbol | Level | | |  |
| --- | --- | --- | --- | --- | --- |
|  |  | Low (-1) | 0 | High (+1) |  |
| Peptone (N- source) | X1 | 10 | 12.5 | 15 | g/L |
| Dextrose (C- source) | X2 | 20 | 25 | 30 | g/L |
| Temperature | X3 | 25 | 28 | 30 | °C |
| Incubation period | X5 | 10 | 15 | 20 | day |
| Inoculum size | X4 | 1 | 2 | 3 | disc per 50 mL |

Table S2. Experimental independent variables at three levels affecting bioactive compounds production isolate using Response Surface design.

| Variables | Symbol | Level | | |  |
| --- | --- | --- | --- | --- | --- |
|  |  | Low (-1) | 0 | High (+1) |  |
| Peptone (N- source) | X1 | 10 | 12.5 | 15 | g/L |
| Dextrose (C- source) | X2 | 20 | 25 | 30 | g/L |
| Temperature | X3 | 25 | 28 | 30 | °C |

Table S3. Binding scores and comprehensive intermolecular interactions of metabolites from the endophytic fungus *M. circinelloides* and the targeted enzyme Tyrosinase.

| Ligand | Binding affinity | Type of bond | Interacting amio acids | H-bond length A |
| --- | --- | --- | --- | --- |
| 3-Methoxy-2,4,6-trimethyl-cyclohex-2-enone | -6.1 | Hydrogen Bond  Hydrogen Bond  Hydrophobic | HIS60  HIS204  HIS208 | 3.0  3.6  3.6 |
| 2-Fluoro-3-trifluoromethylbenzoic acid, 3-hexadecyl ester | -5.9 | Hydrogen  Hydrogen  Hydrogen  Hydrogen  Halogen  Halogen  Halogen  Halogen  Halogen  Hydrophobic  Hydrophobic  Hydrophobic  Hydrophobic | ASN205  ARG209  ARG209  ASN205  ASN205  ASN205  ASN205  ASN205  GLY216  VAL218  VAL218  PRO219  HIS208 | 2.6  2.6  2.3  3.0  3.1  3.2  3.6  3.6  3.2  4.3  3.9  4.3  4.7 |

**Fig. S1. Dose–response curves of the DPPH radical scavenging activity of the optimized EF2 crude extract and ascorbic acid. IC₅₀ values were determined by nonlinear regression analysis using GraphPad Prism. Data are presented as mean ± SD (n = 3).**

**Fig. S2.** Concentration-dependent cytotoxic activity of the optimized EF2 crude extract against A431cell line. A sigmoidal dose–response curve was generated by nonlinear regression analysis to determine the IC₅₀ value. Data are presented as mean ± SD (n = 3).

**Fig. S3.** **Concentration-dependent inhibition of COX-2 by the optimized EF2 crude extract compared with celecoxib. Sigmoidal dose–response curves were generated by nonlinear regression analysis to calculate the IC₅₀ values. Data are presented as mean ± SD (n = 3).**

**Fig. S4. GC–MS chromatogram of** optimized EF2 crude extract **showing the major detected metabolite**
